# Chinese expert concern and consensus on applications of artificial intelligence in clinical cancer imaging

**DOI:** 10.1186/s13244-026-02359-5

**Published:** 2026-07-24

**Authors:** Hong Wu, Xiaorui Yin, Jiejun Cheng, Jiayin Zhang, Linfeng Zheng, Lei Zhang, Feiyun Wu, Qiufeng Zhao, Jun Yang, Han Wang

**Affiliations:** 1 Department of Radiology, Shanghai General Hospital, Shanghai Jiao Tong University School of Medicine, Shanghai, China; 2 Department of Radiology, Shanghai First Maternity and Infant Hospital, Tongji University, Shanghai, China; 3 Department of Radiology, The First Affiliated Hospital With Nanjing Medical University, Nanjing, China; 4 Department of Radiology, Longhua Hospital, Shanghai University of Traditional Chinese Medicine, Shanghai, China; 5 Department of Radiology, Tong Ren Hospital, Shanghai Jiao Tong University School of Medicine, Shanghai, China; 6 Shanghai General Hospital Branch of National Center for Translational Medicine (Shanghai), Shanghai, China

**Keywords:** Artificial intelligence, Neoplasms, Machine learning, Diagnostic imaging, Consensus

## Abstract

**Abstract:**

Artificial intelligence (AI) demonstrates potential throughout the cancer care continuum, with evidence supporting its application in medical imaging for detection, staging, treatment planning, and prognostic evaluation. However, clinical translation is hindered by challenges, data curation and annotation, model interpretability, generalizability, and integration into workflows. To address these barriers and provide guidance, a national multidisciplinary expert panel in China developed this consensus. A modified Delphi approach was employed to achieve expert consensus, involving 81 specialists in radiology, nuclear medicine, oncology, and imaging AI from university hospitals across China. These experts completed a survey containing 30 core statements addressing AI applications in clinical cancer imaging, spanning cancer screening, diagnosis, staging, treatment planning, response assessment, prognostic prediction, data governance, and implementation. Consensus was defined as a mean score ≥ 7 on a 9-point Likert scale, with ≥ 80% of experts scoring ≥ 7. All 30 statements fulfilled these thresholds, with mean scores ranging from 8.06 to 8.58 and the proportion of experts scoring ≥ 7 ranging from 86% to 98%. This expert consensus summarizes key AI application scenarios in cancer imaging and delivers recommendations on data acquisition and annotation, model development and validation, interpretability, multicenter generalizability, privacy-preserving collaboration, clinical workflow integration, and post-deployment monitoring, while contextualizing these statements across major clinical application domains and key implementation challenges in practice. It further identifies priority research directions, including the integration of multimodal and multi-omics data, longitudinal modeling of treatment response, and prospective validation in clinical settings, to support the safe, effective implementation of AI technologies in cancer imaging.

**Key Points:**

- **Question** AI translation in oncologic imaging remains constrained by limitations in rigorous validation, actionable interpretability, standardization, governance, and workflow integration.
- **Findings** Eighty-one Chinese experts reached consensus on 30 clinically practical statements covering AI applications from early detection to deployment.
- **Critical relevance statement** Recommendations highlight expert-supervised labeling, multicenter validation, subgroup evaluation, interpretable outputs, privacy-secured collaboration, integrated workflows, and post-implementation surveillance.

## Introduction

The global incidence and mortality of cancer have been steadily rising, establishing cancer as one of the foremost threats to human health. According to the Global Cancer Observatory (GLOBOCAN), there were 20 million new cancer cases and 9.7 million cancer-related deaths worldwide in 2022. Projections indicate that by 2050, the annual number of new cancer cases will exceed 35 million globally, representing a 77% increase compared to 2022 [1]. In China, GLOBOCAN estimated nearly 4.8 million new cancer cases in 2022, an increase from 2020 [2], along with approximately 2.6 million cancer-related deaths. Lung, liver, stomach, colorectal, and esophageal cancers were the leading causes of cancer mortality in the country. While the overall 5-year survival rate for cancer in China has shown a consistent upward trend [3], this improvement is likely attributed to advances in early diagnosis, precision treatment, and enhanced access to healthcare services [4]. Nevertheless, conventional approaches to cancer diagnosis and treatment face significant limitations in accuracy, efficiency, and personalization. These include difficulties in detecting early-stage occult tumors, differentiating diseases with overlapping clinical presentations, managing treatment-resistant malignancies and post- therapeutic recurrence risks, and implementing fully individualized therapeutic strategies [5].

Over the past decade, artificial intelligence (AI) techniques, particularly radiomics and deep learning (DL), have been extensively investigated for applications in cancer screening, diagnosis, staging, treatment planning, response assessment, and prognostic prediction [6–11]. A growing body of feasibility and early-phase clinical studies has demonstrated that AI has the potential to improve lesion detection, reduce inter-observer variability, and extract quantitative imaging biomarkers capable of capturing tumor biology and heterogeneity [7–11]. However, the majority of AI models have been evaluated only in retrospective or experimental research settings, and robust evidence supporting their routine integration into clinical practice remains limited.

Several barriers contribute to this translation gap. These include heterogeneity in imaging acquisition protocols, annotation methodologies, modeling pipelines, and validation frameworks, all of which collectively impair reproducibility and external generalizability [12]. Many AI models function as “black boxes,” offering limited interpretability for clinicians. This lack of transparency may compromise clinical accountability, hinder transparent communication with patients, and obscure potential algorithmic biases, thereby raising significant ethical concerns [13]. Furthermore, integration of AI tools into clinical workflows, including picture archiving and communication systems (PACS), radiology information systems (RIS), and multidisciplinary decision-making processes, remains suboptimal. In addition, persistent challenges related to data privacy, secure inter-institutional data sharing, and continuous monitoring of model performance in real-world settings continue to impede broad clinical adoption.

In China, the growing cancer burden is placing increasing demands on scalable and standardized oncologic imaging workflows, as well as on imaging-based clinical decision-support systems. Meanwhile, research in imaging AI has expanded in scope and complexity, driven by expanding multicenter collaborations and translational initiatives. However, clinicians, radiologists, and data scientists continue to face practical uncertainties regarding the appropriate clinical workflows for AI integration, the standardization of data acquisition and annotation protocols, the requisite levels of validation prior to deployment, and strategies for balancing innovation with risk mitigation. These challenges extend beyond the scope of individual studies and underscore the need for coordinated, multidisciplinary expert consensus to guide responsible implementation.

**Fig. 1:**
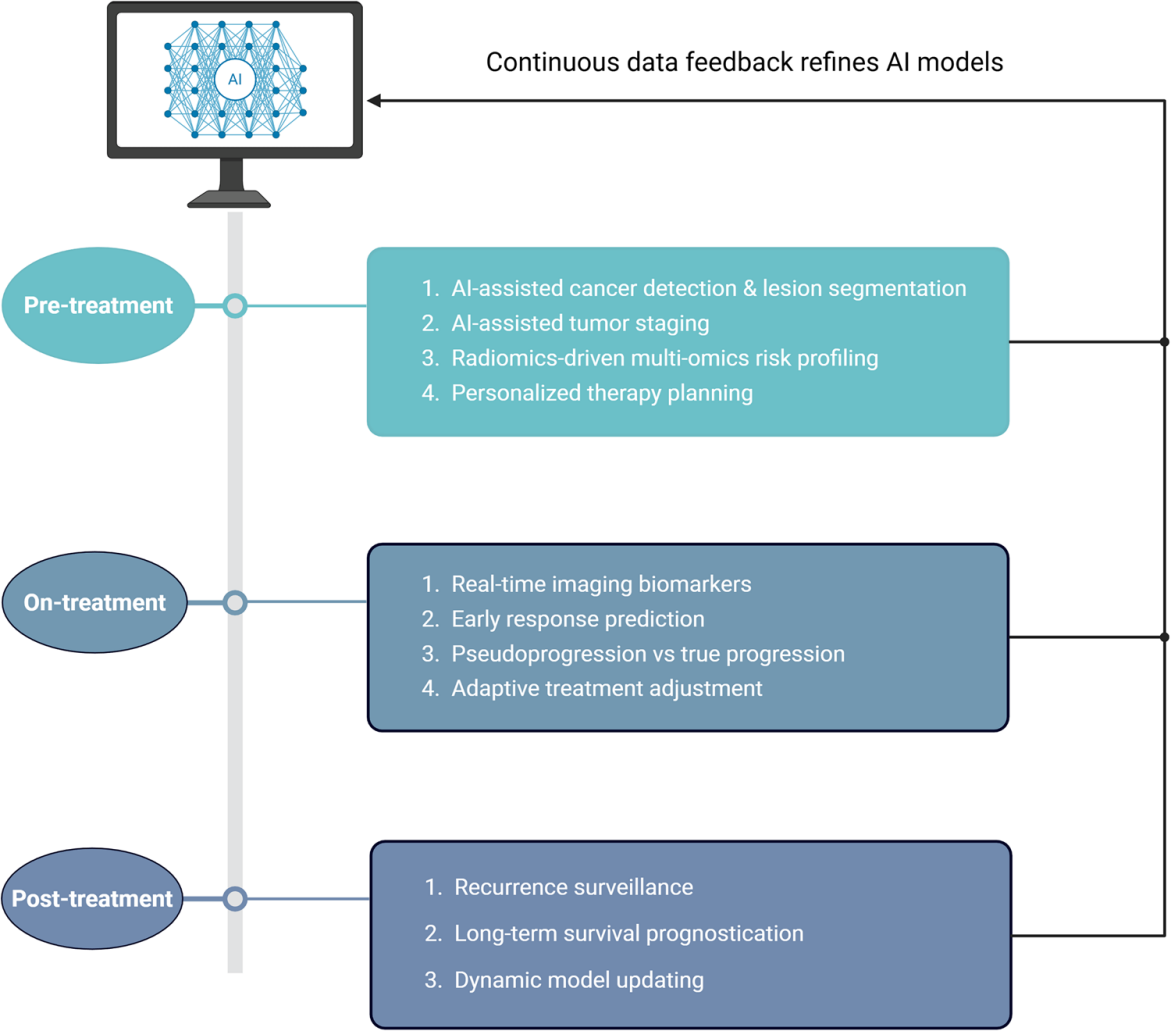
AI-driven closed-loop management across the pre-, intra-, and post-treatment phases

**Fig. 2:**
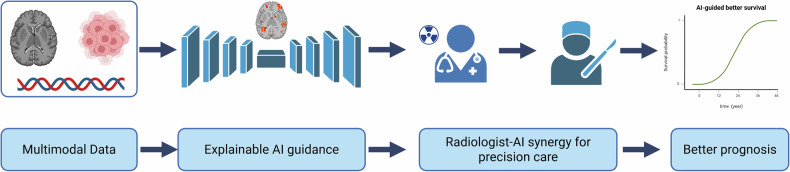
End-to-end integration of AI in cancer imaging from scan to potentially improved survival

To address these gaps, a national multidisciplinary expert panel was convened under the auspices of major medical imaging societies in China. Using a modified Delphi methodology with an anonymized electronic survey, the panel evaluated a comprehensive set of statements addressing AI applications in cancer imaging, from early screening to prognostic prediction, as well as key issues related to data quality, standardization, and clinical implementation. Based on the high level of agreement across 30 core statements, this expert consensus was developed. It summarizes current applications of AI in clinical cancer imaging (Fig. 1), provides recommendations covering data governance, model development and validation, interpretability, and clinical workflow integration (Fig. 2), outlines priority research directions to support the safe, effective, and sustainable implementation of AI technologies, and further contextualizes these recommendations through an interpretive discussion of major application domains, translational challenges, and implementation considerations.

## Methods

### Governance and expert panel

This consensus was developed under the coordination of four professional organizations: the Medical Imaging Alliance of the Yangtze River Delta region, the Radiology Department Association of Hospitals of Shanghai, the Medical Imaging Committee of the Shanghai Association of Chinese Integrative Medicine, and the Molecular Imaging Group of the Chinese Society of Radiology. A steering committee was responsible for overseeing the methodological design, development of consensus statements, and final approval of the document.

Panel experts were selected based on their clinical experience in oncologic imaging, academic contributions to radiomics and AI, and leadership roles within regional or national professional societies. The expert panel comprised radiologists, nuclear medicine physicians, oncologists, and researchers from university hospitals and academic institutions across China. The steering committee members are listed in Supplementary Table S1.

### Development of consensus statements

The preliminary set of consensus statements was developed by the steering committee and the core writing group. To ensure comprehensive coverage of key themes in AI for cancer imaging, the writing group conducted a targeted literature review and synthesized evidence on AI applications across the cancer care continuum, including screening, diagnosis, staging, treatment planning, response assessment, and prognostic modeling. Cross-cutting considerations such as radiomics, model interpretability, privacy protection, data standardization, and clinical implementation were also systematically summarized. The literature review was conducted in PubMed, encompassing studies indexed up to November 2025, using combinations of keywords related to cancer, imaging modalities, and AI methodologies (e.g., machine learning (ML), DL, radiomics). Human studies focusing on clinically relevant imaging tasks were prioritized; Non-oncology or non-imaging studies, purely technical papers lacking clinical evaluation, and opinion-only articles were excluded. Full texts were independently screened by two reviewers, with discrepancies resolved through consensus discussion. This review aimed to identify key application scenarios and critical implementation challenges to inform the drafting of consensus statements. Based on this process, the writing group formulated an initial pool of candidate statements that reflected current challenges, emerging opportunities, and domains requiring expert agreement.

The writing group refined the draft statements through multiple rounds of internal reviews to ensure clarity, clinical relevance, and freedom from redundancy. The finalized statements were subsequently circulated to the expert panel for independent evaluation via the Delphi survey. The final questionnaire comprised thirty consensus statements addressing early cancer detection, diagnostic assessment and lesion characterization, staging and treatment planning, treatment response and prognostic prediction, as well as key cross-cutting issues related to data standardization, privacy protection, and clinical implementation.

### Delphi procedure

A modified Delphi method was used, employing an anonymized electronic survey administered in November 2025. Experts independently rated each statement on a 9-point Likert scale (1 = strongly disagree; 9 = strongly agree) and were invited to provide optional free-text comments. Anonymity was preserved throughout the process to minimize dominance bias and ensure equitable representation of expert opinions. Descriptive statistics were used to summarize the rating distribution for each statement. No group-level feedback was provided during the voting round to maintain independent judgment.

### Consensus criteria and decision rules

Consensus was predefined as a mean score ≥ 7.0 with at least 80% of experts assigning a score ≥ 7. Statements that did not meet these thresholds were designated for revision and scheduled for re-evaluation in subsequent Delphi rounds. Finalized statements were subject to review and formal endorsement by the steering committee prior to inclusion in the consensus document.

### Ethical considerations

This initiative was based solely on expert opinion and did not involve human participants or the use of patient data. Institutional review board approval was therefore waived. All panel members provided consent for the anonymized analysis and aggregation of their responses.

## Results

### Delphi outcomes

The Delphi panel consisted of 81 experts. The majority were radiologists (62/81, 76.54%), followed by nuclear medicine physicians (8/81, 9.88%), oncologists (6/81, 7.41%), and data science or AI-related specialists (2/81, 2.47%). With respect to clinical seniority, 79/81 (97.53%) were attending-level physicians. Overall, 75/81 (92.59%) reported at least 10 years of relevant experience, including 49/81 (60.49%) with more than 20 years. Primary practice and research domains, allowing multiple selections, were predominantly in oncologic imaging diagnosis (61/81, 75.31%) and medical imaging AI and radiomics research (44/81, 54.32%). Additional areas of expertise included molecular imaging in oncology (16/81, 19.75%), imaging-guided therapy or radiotherapy (15/81, 18.52%), clinical oncology care (11/81, 13.58%), and multi-omics and translational medicine (6/81, 7.41%). Most panel members were affiliated with university or academic hospitals (68/81, 83.95%), with additional representation from regional hospitals (8/81, 9.88%), research institutes (4/81, 4.94%), and other institutions (1/81, 1.23%). Geographically, the panel represented at least nine cities across China, including major metropolitan centers such as Beijing and Shanghai.

All thirty statements met the predefined consensus criteria in the first Delphi round. Mean agreement scores ranged from 8.06 to 8.58, and the proportion of ratings ≥ 7 ranged from 86% to 98% across all statements. No statements required revision, and no additional Delphi rounds were necessary.

### Final consensus statements

The finalized consensus statements on the clinical application of AI in cancer imaging are summarized in Table 1. These statements are organized into five thematic categories: early cancer screening, diagnostic assessment and lesion characterization, staging and treatment planning, treatment response assessment and prognostic prediction, as well as data standardization, clinical implementation, and future research directions.

**Table 1:** Final consensus statements on the clinical application of artificial intelligence in cancer imaging

**Early cancer screening**
• Artificial intelligence (AI)–based models that have undergone adequate validation can be used as decision-support tools to assist radiologists in cancer screening, with the aim of improving screening efficiency and sensitivity.
• The development of diagnostic AI models should encourage the integration of multimodal imaging data to enhance model robustness and diagnostic accuracy.
**Diagnostic assessment and lesion characterization**
• In oncologic imaging research, automated or semi-automated segmentation methods, combined with expert review, are preferred to reduce subjectivity and improve reproducibility.
• For tasks such as diagnosis, subtype classification, and risk prediction, AI models should integrate radiomics features with deep learning–derived features to improve stability and biological relevance.
• In settings with limited data availability, rare diseases, or insufficient annotations, strategies such as transfer learning, weakly supervised learning, or few-shot learning may be adopted to improve model feasibility and performance.
• AI models used for oncologic imaging diagnosis should provide interpretable outputs sufficient to support clinical understanding of model focus, decision rationale, potential failure modes, and human expert review or override when clinically appropriate.
**Staging and treatment planning**
• In tumor staging, AI can serve as an assistive tool but should not replace comprehensive clinical judgment in the absence of sufficient prospective evidence.
• AI models for tumor staging or risk assessment should incorporate structural, functional, and other multimodal imaging information to improve the detection of occult or subtle lesions.
• During preoperative planning, automated image analysis methods may be used to identify tumors and surrounding critical structures in order to achieve stable and consistent assessment.
• In radiotherapy-related segmentation tasks, AI tools may be used under professional supervision to assist in the delineation of target volumes and relevant anatomical structures, thereby improving consistency and efficiency.
• Treatment recommendation and outcome prediction models should integrate imaging features, clinical information, and other multi-source data to support individualized treatment decision-making.
• AI models used in surgical navigation or intelligent surgical assistance systems should undergo thorough validation of safety, accuracy, and stability prior to clinical deployment.
**Treatment response assessment and prognostic prediction**
• In complex therapeutic settings, such as immunotherapy, AI models may assist in distinguishing true disease progression from treatment-related imaging changes, thereby reducing the risk of misinterpretation.
• Treatment response assessment should incorporate longitudinal, multi–time point imaging information to more comprehensively reflect tumor dynamics.
• Prediction models for treatment response may combine structural and functional imaging data to enhance the identification of therapeutic effects.
• Intratumoral heterogeneity and peritumoral microenvironmental features may be incorporated into AI models to improve prognostic prediction accuracy.
• AI models intended for prognostic assessment should undergo external validation across centers, imaging devices, patient populations, and clinically relevant subgroups prior to clinical adoption, with attention to potential performance disparities.
**Data standardization, implementation, and future directions**
• Oncologic imaging AI research should strive to harmonize imaging acquisition parameters and preprocessing pipelines to mitigate the impact of inter-institutional variability.
• Automated or semi-automated annotation approaches, combined with expert review, are recommended to improve annotation consistency and efficiency.
• In multicenter collaborations, privacy-preserving collaborative modeling techniques may be prioritized to avoid direct sharing of raw imaging data.
• When applying privacy-preserving techniques, their potential impact on image quality and model performance should be carefully evaluated to avoid degradation of diagnostic capability.
• AI models intended for clinical use should provide an appropriate level of interpretability to enhance transparency and clinical trust.
• AI tools should be deeply integrated with clinical information systems, such as picture archiving and communication systems and radiology information systems, to enable actionable clinical workflows.
• Prior to clinical deployment, physicians should receive appropriate training to understand the capabilities, limitations, and risks associated with AI tools.
• Before entering routine clinical practice, AI tools should be evaluated through prospective studies or real-world evidence to verify their actual clinical value.
• In primary or resource-limited settings, lightweight AI tools may be preferentially deployed to improve accessibility and operational feasibility.
• Future research should promote integrated modeling of imaging, pathology, genomic, and other multi-omics data.
• In rare cancers or data-limited scenarios, strategies such as transfer learning, multicenter collaboration, or cross-cancer knowledge transfer should be considered.
• Dynamic regulatory and quality control mechanisms should be established to monitor safety and stability over time, data shift, performance drift, and performance in relevant patient subgroups after deployment.
• The scope of application, target populations, and limitations of AI models should be clearly defined to avoid inappropriate generalization or misuse.

Collectively, these statements reflect expert agreement on the appropriate roles of AI across the cancer imaging workflow, key methodological considerations for model development and validation, and essential requirements for safe and effective clinical adoption.

### Consensus interpretation

This section interprets the consensus statements by organizing them into major domains of clinical application and implementation in cancer imaging. The expert panel agreed that AI shows particular promise in areas such as early detection and screening, lesion segmentation and characterization, staging and treatment planning, and treatment response assessment and prognostic prediction. At the same time, the panel emphasized that translation into routine practice requires robust supporting evidence, including prospective evaluation, multicenter external validation, and real-world performance monitoring.

### Cancer screening and diagnostic assessment

For early detection and screening, the consensus supports the use of adequately validated AI models as decision-support tools to assist radiologists in improving screening efficiency and sensitivity. In this context, accumulating evidence suggests that AI-assisted image interpretation can improve workflow efficiency while maintaining diagnostic performance comparable to conventional expert-based workflows. Prospective evaluations in population-based screening mammography have demonstrated that AI systems, when implemented as an independent second reader within paired-reader non-inferiority study designs, can achieve cancer detection rates non-inferior to those of dual radiologist readings, while improving workflow efficiency and accessibility, particularly in settings with limited radiology resources [14, 15]. Similar findings have been reported for DL models applied to non-contrast computed tomography (CT), where AI-based analysis of routinely acquired images has enabled sensitive detection of malignancies such as pancreatic cancer, supporting the feasibility of opportunistic screening approaches in resource-constrained environments [16].

The consensus further emphasizes that diagnostic AI development should encourage the integration of multimodal imaging data, and that for core clinical tasks, including diagnosis, molecular subtype classification, and risk prediction, it explicitly advocates combining radiomics features with deep learning–derived features to enhance model stability, generalizability, and biological interpretability. Moreover, beyond screening applications, noninvasive imaging-based assessment holds demonstrable clinical utility as a complementary alternative to invasive tissue sampling in carefully selected clinical scenarios, particularly where biopsy is contraindicated, technically challenging, or carries unacceptable morbidity. Multicenter studies have shown that the DL model ThyNet may differentiate benign from malignant thyroid nodules on ultrasound, and may help reduce unnecessary biopsies without compromising diagnostic accuracy [17]. Likewise, studies integrating MRI features with genomic data for glioma grading [18], or combining CT-based radiomics with liquid biopsy for the noninvasive differentiation of gallbladder cancer [19], illustrate the potential of AI to decode radiological phenotypes into biologically meaningful insights, thereby supporting precision oncology and the characterization of tumor heterogeneity [11].

Automated or semi-automated segmentation methods, particularly when integrated with expert review, may help reduce subjectivity and improve reproducibility in oncologic imaging research. Accordingly, standardized, auditable lesion segmentation and feature extraction constitute foundational, non-negotiable steps for robust downstream diagnostic modeling, prognostic prediction, and quantitative treatment response assessment. Manual region-of-interest annotation is labor-intensive and susceptible to inter-observer variability, limiting reproducibility and scalability in clinical translation [20–22]. In this context, automated and semi-automated DL–based segmentation approaches may improve consistency and efficiency. Methods based on U-Net architectures and their variants, such as nnU-Net [23], have shown robust performance in tumor region extraction and may reduce reliance on manual labeling while helping to minimize inter-observer variability [24, 25].

The consensus further affirms the critical value of multimodal integration and data-efficient learning strategies, such as self-supervised pretraining, few-shot adaptation, and synthetic data augmentation, for lesion characterization, especially in resource-constrained settings characterized by limited annotated datasets, rare tumor subtypes, or sparse clinical ground truth. By synergistically fusing complementary structural (e.g., CT, MRI) and functional (e.g., PET, DCE-MRI, DWI) imaging information, multimodal approaches demonstrably enhance lesion delineation accuracy, differential diagnostic confidence, and biologically informed characterization. For example, in hepatocellular carcinoma, metabolic information from PET may facilitate lesion delineation when CT findings are ambiguous, whereas CT may assist in localizing low-metabolism necrotic regions identified on PET. However, current approaches still often lack effective modeling of cross-modality interactions and resolution-specific features, making PET–CT fusion an active area of ongoing research aimed at further improving hepatic lesion segmentation [26]. In parallel, weakly supervised learning and transfer learning approaches have shown feasibility for automated segmentation in data-limited scenarios such as pediatric medulloblastoma, supporting the corresponding consensus statement on rare cancers and limited data settings [27].

### Cancer staging and treatment planning

In the consensus statements on staging, AI is affirmed as a clinically valuable assistive tool for enhancing both inter-rater consistency and anatomical granularity in TNM-based tumor assessment, particularly for challenging determinations such as extracapsular extension in head and neck cancer, microscopic vascular invasion in hepatocellular carcinoma, and nodal metastasis characterization across modalities. Conventional staging systems rely heavily on visual image interpretation and are susceptible to inter-observer variability. In contrast, AI models can extract quantitative features from multimodal imaging modalities, including CT, MRI, and PET–CT, to support more standardized assessment of primary tumor extent, nodal involvement, and distant metastasis (DM). Evidence supporting AI-assisted T staging has been reported across several tumor types. In rectal cancer, early DL models focused on binary classification of tumor depth, whereas more recent multiplanar frameworks (e.g., Faster R-CNN–based models integrating axial, sagittal, and coronal T2-weighted MRI) have enabled discrimination across the full spectrum of T1–T4 stages, thereby improving staging resolution and reproducibility [28–30].

The consensus also highlights lymph node assessment and DM prediction as key areas in which AI may provide additional value for staging and treatment decision-making. Accurate assessment of nodal status remains a critical determinant of treatment planning and prognosis. In early-stage non–small cell lung cancer (NSCLC), occult mediastinal lymph node metastases are detected intraoperatively in a subset of patients, often necessitating unplanned modifications to adjuvant treatment strategies [31–35]. The consensus highlights that AI models integrating morphological lymph node features with metabolic information from PET–CT may help overcome the limitations of size-based criteria. For example, a Faster R-CNN–based model applied to MRI has been reported to enable rapid and accurate detection of lymph node metastases, with reduced interpretation time compared to manual assessment [36]. Similarly, CT-based AI models have demonstrated the feasibility of noninvasive prediction of occult nodal metastasis in laryngeal cancer, potentially supporting risk-adapted surveillance strategies [37].

The panel also agreed that AI has potential value in the early detection and prediction of DM, which is essential for optimizing outcomes in advanced cancer [38]. While whole-body imaging plays a central role in metastatic evaluation [39–41], interpretation of modalities such as bone scintigraphy remains challenging due to limited spatial resolution and subjective interpretation. End-to-end multi-task DL approaches (e.g., U-Net–based architectures) have been explored to predict clinical outcomes directly from whole-body imaging data, such as bone scintigraphy, thereby enabling automated lesion detection and anatomical localization [42]. Notably, several studies suggest that AI models may predict DM using features derived solely from primary tumor imaging. Radiomics-based models (e.g., an MRI radiomic signature constructed using LASSO–Cox regression and incorporated into a radiomic nomogram) have demonstrated feasibility in predicting DM and guiding adjuvant chemotherapy decisions in locally advanced rectal cancer [43]. A CT-based DL radiomics model using a pre-trained DenseNet feature extractor and a random-forest classifier, combined with clinical information, has been shown to predict DM in breast cancer and to identify network-derived signatures as potential biomarkers [44].

However, the consensus also underscored that fully automated, tumor-specific TNM staging systems are not yet ready for routine clinical use. Current limitations include dependence on training data quality and scale, labor-intensive annotation processes, heterogeneity across multicenter datasets, limited interpretability of model outputs, and a predominance of retrospective, single-center validation studies, along with unresolved ethical and regulatory challenges [45–47]. Accordingly, AI should currently be positioned primarily as an assistive tool for staging, and broader clinical integration will require prospective validation, improved interpretability, and more robust multicenter evidence.

In the consensus statements related to treatment planning, AI was considered relevant to both local treatment planning, particularly in surgical oncology, and systemic therapy selection. The growth of AI-related research in surgical oncology over recent decades reflects increasing interest in its potential to support preoperative planning and intraoperative guidance [48, 49]. AI-based image analysis may assist in delineating tumor boundaries and their spatial relationships with surrounding anatomical structures, thereby supporting more precise surgical planning [50].

However, the expert panel noted that manual segmentation remains inefficient and prone to inconsistency, underscoring the need for automated approaches in surgical planning and intraoperative guidance [51]. At present, the maturity of AI-based segmentation varies substantially across different application targets. While AI has demonstrated relatively high and stable performance in organ-level segmentation, its accuracy for solid tumor segmentation remains variable, with performance gaps documented in international benchmarking challenges [50]. Within this context, advances in vascular segmentation using V-Net–based algorithms represent a comparatively more mature application, potentially enabling improved preoperative assessment of tumor–vessel relationships, particularly in hepatic interventions [52]. Similarly, intraoperative AI-assisted navigation and super-resolution imaging techniques have demonstrated potential to enhance surgical visualization and procedural safety in selected clinical settings [53, 54]. Augmented reality–integrated systems have been shown to achieve high segmentation accuracy and submillimeter navigation error in specific surgical scenarios, thereby supporting individualized surgical planning and margin control under well-defined conditions [55]. These findings support the potential of AI-assisted surgical planning, but not yet uniform readiness for widespread clinical deployment.

In the context of chemotherapy and targeted therapy, the consensus highlights AI-driven treatment recommendation systems that integrate radiomics, genomics, and electronic health record (EHR) data to support personalized therapy selection. By analyzing pre- and post-treatment imaging features in conjunction with molecular biomarkers, AI models may assist in predicting therapeutic response and minimizing overtreatment. For example, DL–derived imaging scores from ^18^F-FDG PET/CT have demonstrated the ability to noninvasively predict epidermal growth factor receptor mutation status in NSCLC, thereby informing the selection of targeted versus immunotherapeutic regimens [56]. Similarly, multimodal Transformer-based models integrating radiological, pathological, and clinical data have shown promise in predicting responses to targeted and immunotherapeutic strategies in human epidermal growth factor receptor 2-positive gastric cancer [57]. Overall, these studies support the consensus view that AI may enhance treatment planning across both local and systemic contexts, although continued methodological refinement and prospective clinical validation remain necessary before routine adoption.

### Treatment response assessment and prognostic prediction

In the consensus statements related to treatment response assessment, AI was regarded as a potentially useful adjunct to conventional imaging criteria, particularly in clinical settings where static size-based evaluation may be insufficient. The Response Evaluation Criteria in Solid Tumors (RECIST) version 1.1 remains the most widely used standard for evaluating treatment response in solid tumors [58, 59]. However, the expert panel agreed that RECIST-based size measurements have inherent limitations when applied to immune checkpoint inhibitor–based therapies. In particular, pseudoprogression (PP)—a transient increase in tumor size followed by subsequent regression—may be misclassified as true disease progression, potentially leading to premature discontinuation of effective treatment [60, 61].

To address these challenges, immune-specific response criteria, including immune-related response criteria (irRC) [62], immune-related RECIST (irRECIST) [63, 64], and immune RECIST (iRECIST) [65], have been proposed. Although iRECIST introduces the concept of unconfirmed progression, the consensus noted that quantitative standards for dynamic changes in non-target lesions remain insufficient. Moreover, definitive differentiation between PP and true progression often relies on biopsy, which is limited by procedural invasiveness and sampling variability [66].

Against this background, the consensus supports the development of noninvasive AI-based approaches for dynamic and quantitative response assessment. By extracting high-dimensional features from tumor-related regions of interest, radiomics-based models have demonstrated feasibility in predicting hyperprogressive disease and treatment response in patients with NSCLC receiving immunotherapy [66], as well as in forecasting post-radiosurgical PP and long-term tumor outcome following stereotactic radiosurgery for vestibular schwannoma [67]. These studies provide evidence that AI-driven imaging analysis may support dynamic, data-informed adjustment to treatment strategies across multiple tumor types.

The consensus also highlights multimodal and longitudinal integration as important directions for improving response assessment. Models incorporating radiomic features from PET, CT, and fused PET/CT images have been shown to improve predictive performance for treatment efficacy in advanced NSCLC [68]. Similarly, models integrating clinical variables with CT–derived radiomic features have demonstrated moderate-to-high accuracy in predicting response to immune checkpoint blockade [69]. In metastatic melanoma, noninvasive PET/CT radiomics combined with blood-based parameters have emerged as promising biomarkers for early differentiation between PP and true progression, potentially reducing unnecessary toxicity or delayed treatment modifications [70].

The panel further noted emerging evidence supporting comprehensive multimodal strategies that integrate imaging, molecular, and clinical data. For example, models combining longitudinal MRI–based spatial habitat radiomics with transcriptomic and single-cell RNA sequencing data have demonstrated feasibility in predicting pathological complete response in breast cancer, with model outputs correlated with patterns of immune cell infiltration [71]. These findings are consistent with the corresponding consensus statements, while also indicating that such approaches remain exploratory and require further prospective validation before routine use.

In the consensus statements related to prognostic prediction, AI was viewed as a promising tool for individualized risk stratification beyond conventional population-level estimates. Tumor staging systems such as TNM remain indispensable for prognosis assessment due to their simplicity and broad clinical applicability [72]. Nevertheless, limitations of traditional staging frameworks have been increasingly recognized in the era of precision oncology, prompting calls for AI-assisted approaches to reduce inter-observer variability and enhance individualized risk stratification [73].

By integrating multidimensional imaging and clinical data, AI has the potential to shift prognostic assessment from population-level estimates toward personalized outcome prediction. Studies have suggested that CT-based radiomics may predict early recurrence of intrahepatic cholangiocarcinoma and support survival prediction when combined with clinical variables [74]. Similarly, models integrating MRI–derived radiomic features with DL architectures have suggested potential in predicting occult cervical lymph node metastasis in early-stage oral and oropharyngeal squamous cell carcinoma, thereby informing surgical decision-making and potentially avoiding overtreatment in a substantial proportion of patients [75].

The consensus further emphasizes expanded feature extraction and multimodal integration as key directions in prognostic modeling. Multimodal frameworks incorporating immune-related long noncoding RNA features, radiomic features, and clinical factors in glioblastoma have demonstrated superior predictive performance compared with unimodal approaches, with radiomic signatures reflecting immune cell infiltration and characteristics of the tumor immune microenvironment [76]. In high-grade serous ovarian cancer, prognostic models based on peritumoral radiomic features have achieved improved prediction of response to platinum-based chemotherapy, underscoring the value of habitat analysis and peritumoral region characterization [77].

Habitat-based radiomics has emerged as a recurrent methodological theme across prognostic studies. For example, K-means clustering–based habitat models using preoperative CT data have been developed to predict postoperative recurrence risk in non–muscle-invasive bladder cancer, with imaging-derived scores correlated with tumor stromal ratio [78]. In addition, nomograms integrating clinical variables with tumor subregional radiomic features have shown promising performance in predicting immune-related adverse events [79]. Overall, these studies support the consensus view that AI may strengthen prognostic assessment through more individualized and biologically informed risk modeling, although broader clinical adoption will still depend on robust validation, interpretability, and workflow-level evaluation.

### Methodological and translational challenges

In the consensus statements related to data standardization, privacy protection, interpretability, and clinical translation, the expert panel identified data availability and annotation as foundational barriers to the development and deployment of robust AI models in cancer imaging. Training supervised AI models typically requires large volumes of high-quality labeled data, yet access to comprehensive and representative datasets in oncology remains limited. In many tumor-related applications, pathological findings are used as reference standards; however, obtaining such labels is costly and invasive, and often constrained by limited availability and preservation challenges. Moreover, prognostic differences between patients who undergo biopsy and those managed without intervention during screening may introduce systematic bias into model training. In addition to selection bias, oncologic imaging datasets collected in clinical practice may contain imbalances related to institutions, demographic characteristics, disease spectrum, and image acquisition. These imbalances may lead to uneven model performance across centers, scanners, tumor subtypes, or clinically relevant patient groups. Therefore, model validation should include assessment in relevant patient subgroups whenever feasible, together with overall performance metrics.

Manual image annotation, which remains common in many studies, is labor-intensive and inherently subjective, leading to reduced reproducibility and scalability of AI models [80–82]. These challenges are further amplified in multimodal and multi-omics modeling, where increasing the diversity of input data may enhance model expressiveness but simultaneously reduce sample size, increase complexity, and limit generalizability. The consensus, therefore, emphasizes the need for methodological strategies that balance data richness with feasibility and robustness.

Even when AI models demonstrate strong performance during internal validation, overfitting remains a persistent concern, often resulting in degraded performance during prospective or external testing [83]. To address limitations in data quantity and diversity, cross-institutional collaboration has been widely advocated [83, 84]. However, the panel recognized that such collaboration introduces substantial challenges related to data privacy, security, and governance.

From a technical perspective, federated learning has emerged as a prominent strategy for collaborative model training without direct data sharing [85]. Nevertheless, heterogeneity in imaging equipment, acquisition protocols, and population characteristics across institutions can impair model convergence and stability. Differential privacy approaches, which protect patient information through noise injection [86, 87], may further obscure clinically relevant features, such as microcalcifications, thereby reducing diagnostic sensitivity [88].

Legal and ethical considerations present additional complexity. Variability in data anonymization requirements across regulatory frameworks, including the European Union General Data Protection Regulation and the United States Health Insurance Portability and Accountability Act, poses compliance risks for cross-border data exchange [89, 90]. Even within national multicenter studies, inconsistent standards among ethics review committees frequently delay data sharing and project implementation [91, 92]. The panel further noted that biological data, such as genomic information, carries inherent and permanent identifiability, increasing the risk of secondary misuse, including genetic discrimination, and resulting in more restrictive sharing practices compared with imaging data [91].

The consensus further identifies model interpretability and generalizability as major prerequisites for safe and scalable clinical translation. Model interpretability remains a central challenge for the clinical adoption of AI in cancer imaging. DL models, in particular, are characterized by high complexity and limited transparency, which conflicts with the requirements of medical decision-making and undermines clinician trust [93, 94]. Even radiomics-based approaches, despite their closer alignment with interpretable features, require careful explanation to ensure clinical relevance. Adequate interpretability ensures radiologists and relevant clinical practitioners can comprehend the model’s core concerns, assess the clinical credibility of its suggestions, spot possible failure modes, and adjust or overrule outputs when clinical circumstances demand. Such criteria carry great weight in expert-monitored clinical workflows, as AI is designed to assist rather than supplant clinical decision-making. Multiple interpretability techniques have been proposed to address these concerns. Feature attribution methods such as SHapley Additive exPlanations have been widely applied to ML models to quantify individual feature contributions [95–97]. For DL architectures, visual explanation techniques including Class Activation Mapping and Gradient-weighted Class Activation Mapping are commonly used to highlight image regions that influence model predictions [98–101]. Text-based and example-based explanation strategies have also been explored for specific clinical contexts. However, the panel agreed that no single interpretability framework has yet been established as a standard in medical image analysis, and interpretability remains an evolving research area [98, 102, 103].

Inadequate generalization was identified as another major barrier to clinical translation. Variability in imaging hardware, acquisition parameters, and patient populations can substantially degrade model performance when applied outside the training domain. More subtly, models optimized for common tumor phenotypes may fail to recognize rare subtypes or mixed pathological features, introducing the risk of systematic misclassification. The consensus, therefore, emphasizes the importance of evaluating robustness across tumor types and subgroups, rather than relying solely on aggregate performance metrics [104]. Taken together, these challenges underscore that progress in cancer imaging AI depends not only on model performance but also on data quality, privacy-aware collaboration, interpretability, and demonstrated robustness across institutions, populations, and disease subtypes.

### Clinical implementation and workflow integration

**Fig. 3:**
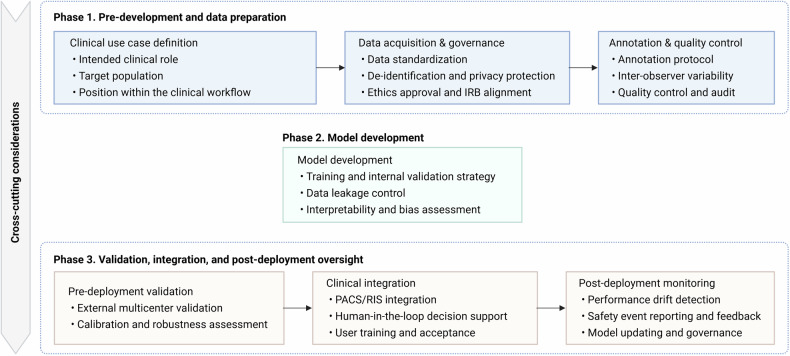
Consensus-based framework across the lifecycle of healthcare AI development and deployment in cancer imaging. The dashed groupings indicate the major phases of the lifecycle. Within each phase, the boxes summarize key domains that should be considered when translating AI tools from development to real-world clinical use, and the arrows indicate the general progression of activities within phases. The vertical sidebar highlights cross-cutting considerations that apply throughout the lifecycle. This framework is intended to guide implementation planning. PACS, picture archiving and communication system; RIS, radiology information system

To facilitate practical translation of these recommendations, Fig. 3 summarizes a consensus-based implementation framework across the lifecycle of healthcare AI development and deployment in cancer imaging. This framework is intended to highlight the major domains that should be considered when moving AI tools from development to real-world clinical use. The expert panel agreed that successful clinical implementation of AI in cancer imaging extends beyond algorithmic performance and requires alignment with real-world clinical workflows. Although academic interest in medical AI continues to grow [105], concerns regarding the potential replacement of physicians persist among clinicians and the public [106, 107]. In practice, however, AI systems are unlikely to function independently and must be embedded within rigorously validated, transparent, and clinically supervised frameworks.

Within this implementation framework, standardization and evidence generation were regarded as core prerequisites for clinical adoption. Translating AI models into routine care requires comprehensive development pipelines, including robust external validation and, where appropriate, prospective or randomized clinical studies [108]. Following clinical implementation, AI tools require ongoing monitoring to detect data shift and performance drift. Such deviations may stem from variations in imaging equipment, scanning protocols, reconstruction algorithms, referral trends, patient demographics, and clinical practice patterns. Once clinically significant performance degradation is identified, standardized protocols shall govern model recalibration, retraining, temporary suspension, or clinical decommissioning. Radiomics research has faced particular scrutiny regarding reproducibility, prompting efforts to standardize components of the radiomics pipeline, such as convolutional filter definitions, to support clinical validation and regulatory approval [109, 110]. Nevertheless, substantial variability remains across key stages of radiomics workflows, including image acquisition, feature extraction and selection, model construction, and validation strategies [111, 112]. The consensus, therefore, emphasizes standardization as a critical priority for advancing clinical adoption [80].

Compatibility with existing clinical information systems and the broader readiness of digital infrastructure were identified as major challenges for implementation. Many AI-based solutions remain poorly integrated with hospital infrastructure, limiting their practical utility [113]. Interoperable, high-performance data communication systems are required to transform heterogeneous imaging, laboratory, pathology, and EHR data into actionable clinical resources [114]. At present, most institutions lack seamless integration between oncology AI tools and core clinical systems.

Successful implementation also depends on human factors and organizational readiness, rather than technology alone. Clinicians often report limited familiarity with AI tools and express concerns regarding loss of decision-making autonomy and potential legal liability associated with AI-assisted errors [113–115]. In addition, the lack of intuitive, user-friendly interfaces hinders adoption in busy clinical environments. Finally, the deployment and maintenance of AI-enabled clinical workflows require sustained investment in information technology infrastructure and specialized personnel, placing additional financial and organizational burdens on healthcare systems [116].

Beyond conventional discriminative AI models, generative AI, such as large language and multimodal foundation models, represents an emerging technology with potential utility in cancer imaging workflows, particularly for documentation and reporting support, information synthesis, and human–AI interaction [117]. However, most current applications remain exploratory and are not yet widely adopted in autonomous clinical roles. Key risks, including hallucinations, privacy and security concerns, bias and fairness, and unclear accountability, necessitate rigorous evaluation, governance, and post-deployment monitoring before routine use. Therefore, while generative AI is not included among the 30 core consensus statements, it is highlighted here as an emerging direction requiring cautious, evidence-based translation.

Taken together, these considerations indicate that successful implementation requires more than model accuracy alone; it also depends on standardization, clinical validation, interoperable infrastructure, human-centered workflow design, and sustained organizational support. Only through systematic standardization, transparent validation, and thoughtful integration into clinical workflows can AI realize its potential as a reliable and sustainable component of cancer imaging care.

## Conclusion

AI is increasingly being applied in cancer imaging across screening, diagnosis, staging, treatment planning, treatment response assessment, and prognostic prediction. Through a modified Delphi process, this expert consensus summarizes current evidence and multidisciplinary expert agreement on the appropriate clinical roles, methodological considerations, and implementation challenges of AI in cancer imaging. By clarifying areas of opportunity and limitation, the consensus aims to support the safe, standardized, and responsible translation of AI into clinical cancer care.

## Supplementary information

Electronic supplementary material

## Data Availability

Not applicable.

## Abbreviations

AI: Artificial intelligence
CT: Computed tomography
DL: Deep learning
DM: Distant metastasis
EHR: Electronic health record
GLOBOCAN: Global Cancer Observatory
iRECIST: Immune response evaluation criteria in solid tumors
irRC: Immune-related response criteria
irRECIST: Immune-related response evaluation criteria in solid tumors
ML: Machine learning
MRI: Magnetic resonance imaging
NSCLC: Non–small cell lung cancer
PET: Positron emission tomography
PP: Pseudoprogression
RECIST: Response evaluation criteria in solid tumors
RNA: Ribonucleic acid
TNM: Tumor-node-metastasis
