# Supplementary material for: Chinese expert concern and consensus on applications of artificial intelligence in clinical cancer imaging
Source: Insights Imaging. 2026 Jul 24;17:193. doi: 10.1186/s13244-026-02359-5 (PMC13400512; doi:10.1186/s13244-026-02359-5)
Supplement: Supplementary file 1 — Electronic supplementary material [file 13244_2026_2359_MOESM1_ESM.pdf]

Chinese Expert Concern and Consensus on Applications of Artificial Intelligence in Clinical Cancer Imaging

Electronic supplementary material

Supplementary Table S1. Steering committee members

Name	Institution	City	Specialty	Years of Professional Experience
Han Wang	Shanghai General Hospital, Shanghai Jiao Tong University	Shanghai	Radiologist	25
Feiyun Wu	Jiangsu Provincial Hospital	Nanjing	Radiologist	30
Liang Wang	Beijing Friendship Hospital, Capital Medical University	Beijing	Radiologist	30
Fei Yu	Shanghai Tenth People’s Hospital, Tongji University	Shanghai	Nuclear medicine physician	25
Hongzan Sun	Shengjing Hospital of China Medical University	Shenyang	Nuclear medicine physician	25
Xuewen Liu	The Third Xiangya Hospital, Central South University	Changsha	Oncologist	20
Qiang Xue	Affiliated Hospital of Nantong University	Nantong	Oncologist	25
He Wang	Fudan University	Shanghai	AI researcher	20
Guang Yang	East China Normal University	Shanghai	AI researcher	25
